# Assessment of safety and quality of fermented milk of camels, cows, and goats sold and consumed in five localities of Burkina Faso

**DOI:** 10.14202/vetworld.2019.295-304

**Published:** 2019-02-22

**Authors:** Hama Cissé, Jean Ulrich Muandze-Nzambe, Namwin Siourimè Somda, Adama Sawadogo, Soungalo Moustapha Drabo, François Tapsoba, Cheikna Zongo, Yves Traoré, Aly Savadogo

**Affiliations:** Laboratory of Applied Biochemistry and Immunology, University Ouaga 1 Pr Joseph KI-ZERBO, 03 BP 7021 Ouagadougou 03, Burkina Faso

**Keywords:** Burkina Faso, camel, cow, fermented milk, goat, sanitary quality

## Abstract

**Background and Aim:**

Fermented milk is food produced and consumed all over the world and plays an important role in human nutrition. This work aimed to evaluate the microbiological and physicochemical quality and mineral composition of fermented milk consumed in Burkina Faso.

**Materials and Methods:**

A total of 114 samples of fermented milk from camels, goats, and cows were purchased in the market in five localities in Burkina Faso; Bobo Dioulasso, Djibo, Dori, Gorom-Gorom, and Sebba. Microbiological and physical parameters were monitored using standards methods.

**Results:**

Microbiological analysis of fermented milks showed high average values of 7.60±1.50×10^9^ colony-forming unit per milliliter (CFU/ml), 5.72±3.60×10^7^ CFU/ml, 5.53±2.00×10^5^ CFU/ml, 1.97±0.18×10^3^ CFU/ml, 1.98±0.25×10^3^ CFU/ml, and 0.10±0.09×10^3^ CFU/ml for total microbial flora, lactic acid bacteria, yeasts and molds, *Staphylococcus aureus*, total coliforms, and thermotolerant coliforms, respectively. None of the samples were contaminated by *Salmonella* or *Shigella*. The average values of pH, acidity, dry matter, ash, fats, proteins, and total carbohydrates content of samples were ranged, respectively: 3.830-4.137, 1.888-2.822%, 8.271-13.004%, 0.199-0.476%, 1.210-3.863%, 2.125-3.764%, and 3.080-5.428 % (w/w). Na/K and Ca/Mg ratio ranged from 0.104 to 0.909 and from 3.392 to 16.996, respectively. Total microbial flora, yeasts and molds, total coliforms, fats, calcium, potassium, iron, and zinc were significantly different.

**Conclusion:**

This research contributed in the evaluation of the hygienic and nutritional qualities of local fermented milk. Results obtained in this study confirm the need to set up the training program on the sanitary condition to traditional maker’s to ensure the good fermented milk with high organoleptic and nutritional qualities.

## Introduction

Milk is the natural product of the secretion of the mammary gland of a lactating female. It is an essential component of the diet of pastoral or agropastoral populations and also an important source of income in Sahelian countries. Milk plays an important role in bone growth, maintaining body integrity and health through its composition of minerals, fats, proteins, carbohydrates and vitamins [1]. Milk microbiota contains many bacteria, some are useful and necessary for her transformation to other products as lactic acid bacteria or molds used for the maturing of cheese and yeasts transforming sugars to alcohol [2-4].

According to composition, raw milk is an ideal medium for the growth of many microorganisms, unlike fermented milk, where there is a predominance of lactic acid bacteria with some contaminants as *Bacillus*, *Staphylococcus*, and *Escherichia coli* [5,6]. Cow milk is the most milk consumed in the world followed by that of goat, camel, buffalo, mare, and donkey. Taste of camel’s milk varies according to the pasture, is appreciated for its anti-infectious, anticancer, antidiabetic, and reconstructive properties in convalescent patients [7,8]. In other countries as Central Asia, mare milk is used to replaces maternal milk for infants. In Africa and particularly in Burkina Faso, for ethnic and cultural reasons only, the milk of sheep, camel, goat, and cows is consumed. The use of unconventional milk (donkey and mare) is culturally important. Consumers of these products attribute to their medicinal and mystical properties during occult practices. Conventionally, the origin of the milk fermentation is correlated to the appearance of nomadic peoples (Fulani). Fermented milk is a traditional remedy used by the old medical sciences of agropastoral communities. In Burkina Faso, there is a greater diversity of dairy products in diets of the populations which include raw milk, fermented milk, pasteurized milk, yogurt, cheese, cream, butter, *gappal, dèguè*, and soap Fulani [9]. In the past, fermented dairy products as yogurt, fermented milks, and cheese have been recognized as foods with undeniable nutritional qualities [10-12]. Recently, a diversity of yogurt (yogurt with Moringa, pineapple, sweetened, and unsweetened) is sold by local producers in Burkina Faso. These foods are perishable and often contaminated by microorganisms, antibiotics, pesticides (insecticides), detergents, and disinfectants [13]. The hygienic quality of milk and dairy products is considered, as one of the major factors limiting their consumption. Other factors influencing the quality of these products include lack of knowledge in good hygiene practices, preservation conditions, and certain chemical additives used.

This work aimed to evaluate the microbiological, physicochemical, and nutritional qualities of fermented milk produced and consumed in Burkina Faso.

## Materials and Methods

### Ethical approval

Ethical approval does not apply to this type of study. Samples of fermented milk were purchased from the vendors and analyzed in our laboratory.

### Sampling

**Figure-1:**
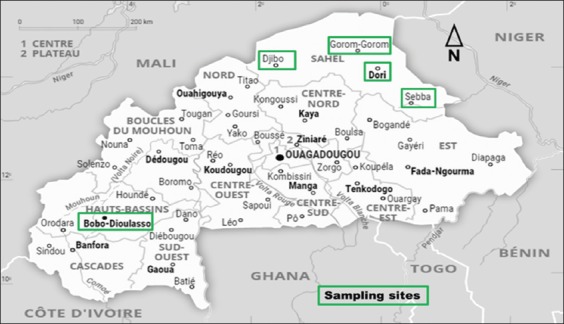
Localities of collected samples (Source: https://www.universalis.fr/atlas/afrique/burkina-faso/#AT003203).

A total of 114 fermented milk samples (camel, cow, and goat) produced by the traditional method, purchased from the markets and streets of five cities in Burkina Faso, were collected aseptically from local producers and transported to the laboratory at 4-5°C using icebox for the different analysis. Figure-1 and Table-1 presented, respectively, sampling sites and samples coding.

**Table-1:** Coding of samples.

Fermented milk	Localities and coding				

	Bobo Dioulasso	Djibo	Dori	Gorom-Gorom	Sebba
Camel	-	*CaJ*	*CaD*	*CaG*	*CaS*
Cow	*CoB*	*CoJ*	*CoD*	*CoG*	*CoS*
Goat	-	*GoJ*	*GoD*	*GoG*	*GoS*

### Microbiological analysis

Microbiological analyses of fermented milk were performed according to standard methods described in the manual of microbiological analysis. The bacterial populations in fermented milk were enumerated after prepared stock solution and decimal dilutions according to standard microbial methods. 10 ml of the sample were added to 90 ml of sterile buffered peptone water, and serial dilutions were monitored with this suspension. All tests were done in duplicate. The results were expressed as colony-forming unit per milliliter (CFU/ml). Total microbial flora was enumerated on plate count agar after incubation at 30°C during 24-48 h. Lactic acid bacteria were enumerated on plates of Man, Rogosa, and Sharpe agar, after incubation at 37°C for 24-48 h anaerobically (anaerobic jars with Anaerocult A). Yeasts and molds were enumerated on Sabouraud CAF agar with chloramphenicol, after incubation at 25°C for 3-5 days. Total coliforms and thermotolerant coliforms were counted on eosin methylene blue agar at 37°C and 44°C for 24-48 h. *Staphylococcus aureus* were counted on Baird-Parker agar supplemented with tellurium egg yolk and incubated at 37°C for 24-48 h, the black brilliant or dark gray colonies surrounded a clear halo were selected and tested for the confirmation (Gram, catalase, and coagulase tests). The research of *Salmonella* or *Shigella* spp. was carried by pre-enrichment with buffered peptone water followed by enrichment in Rappaport-Vassiliadis broth and isolation on *Salmonella-Shigella* agar for 24 h at 37°C after each part.

### Physicochemical analysis

The samples were mixed and analyzed in duplicate for the determination of different parameters physicochemical. The pH was determined using a digital pH meter (WATERPROOF-PC5). Titratable acidity, dry matter, ash, fats, and protein contents were determined according to AOAC [14]. Total carbohydrates were calculated according to this formula: Total carbohydrate = Total solids - (Fat + Protein + Ash) [15].

### Minerals determination

For the determination of mineral elements, the ash was dissolved in 100 ml of concentrated HNO_3_ at 0.5 M. The composition in Ca^2+^, potassium (K^+^), sodium (Na^2+^), magnesium (Mg^2+^), iron (Fe^2+^), and zinc (Zn^2+^) was determined by Fast Sequential Atomic Absorption Spectrometer AA240FS according to AOAC [14]. Table-2 presented the characteristics of analytical curves.

**Table-2:** Characteristics of the calibration curves of minerals.

Mineral	Standard concentration (mg/L)	λ(nm)	Standard solution	Standard gas	Dependence^a^	Correlation coefficient
Ca^2+^	0.0551	422.70	HNO_3_	Air/acetylene	y=0.07993×c	0.9985
Fe^2+^	0.1904	248.20	HNO_3_	Air/acetylene	y=0.02311×c	0.9996
K^+^	0.1497	766.50	HNO_3_	Air/acetylene	y=0.039×c–0.0015	0.9776
Mg^2+^	0.0098	285.20	HNO_3_	Air/acetylene	y=0.45101×c	0.9895
Na^2+^	0.0477	589.00	HNO_3_	Air/acetylene	y=0.09215×c	0.9899
Zn^2+^	0.0499	213.90	HNO_3_	Air/acetylene	y=0.08810×c	0.9889

a y=Flame photometer reading, c=Concentration in mg/L, λ=Wavelength, Ca^2+^=Calcium, K^+^=Potassium, Na^2+^=Sodium, Mg^2+^=Magnesium, Fe^2+^=Iron, Zn^2+^=Zinc

### Statistical analysis

The data were analyzed using analysis of variance by program XLSTAT 2017 and modeled using R software, version 3.4.2 (R Foundation for Statistical Computing, Austria).

The results were expressed as average ± standard deviation. The difference between the means was calculated using least significant difference Fisher’s test, and p<0.05 was considered statistically significant.

## Results

**Figure-2:**
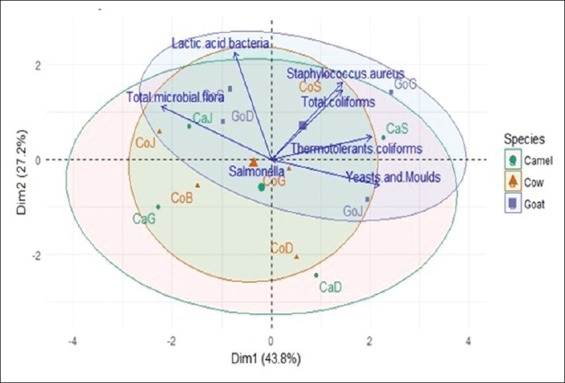
Principal component analysis distribution of fermented milk samples and ellipse of inertia different species on the factorial plane according to microbiological parameter.

The average densities of various microorganisms determined are summarized in Table-3. This result showed a significant load of total microbial flora with high variations of 0.33-7.60×10^9^ CFU/ml. Lactic acid bacteria were found at 0.43-5.72×10^7^ CFU/ml. Yeasts and molds count ranged from 0.33 to 5.53×10^5^ CFU/ml, showing an increasing trend during fermentation. According to the results of this study, total coliform densities were 0.06 at 1.98×10^3^ CFU/ml, and thermotolerant coliform densities ranged from 0.02 to 0.10×10^3^ CFU/ml. *S. aureus* densities were in the range of 0.18-1.97×10^3^ CFU/ml in traditional fermented milk and yogurt sold at acidic pH (>3). Table-3 reveals that *Salmonella* and *Shigella* were absent in all analyzed samples. After analyzing the distribution of centers gravity classes on principal factorial plane (Figure-2), we can be noted some closeness between the types of milk and variables. The variables and samples are visualized in the factorial plane formed on dimensions 1 and 2 (71% of variance explained, Figure-2). According to dimension 1, *CoS* and *GoS* were highly contaminated with total coliforms, *S. aureus*, and lactic acid bacteria, unlike *CaD* and *CoD* which were less contaminated. The dimension 2 reveals that *CaJ*, *CaS*, *CoJ, GoD, GoJ*, and *GoG* were highly contaminated with yeasts and molds and thermotolerant coliforms, but they were weakly contaminated by total microbial flora, while *CaG*, *CoB*, and *CoG* were contaminated with total microbial flora but weakly contaminated by yeasts and molds and thermotolerant coliforms.

**Table-3:** Microbiological parameters of different fermented milk samples.

Samples	TMF×10^9^	LAB×10^7^	Y&M×10^5^	*S. aureus*×10^3^	TC×10^3^	TTC×10^3^	SS
*CaD* (n=6)	0.39±0.27^c^	0.43±0.34^c^	5.44±3.00^a^	0.18±0.06^c^	0.13±0.06^de^	0.06±0.03^ab^	Nd
*CaG* (n=10)	4.08±1.80^b^	2.53±2.37^abc^	0.34±2.70^c^	0.21±0.05^c^	0.06±0.04^e^	0.02±0.01^b^	Nd
*CaS* (n=4)	0.41±0.10^c^	3.10±2.90^abc^	5.53±2.00^a^	1.97±1.18^a^	0.17±0.07^de^	0.08±0.04b^ab^	Nd
*CaJ* (n=4)	4.60±1.39^b^	4.08±3.60^ab^	0.47±0.28^c^	1.23±0.39^abc^	0.08±0.06^e^	0.02±0.00b^ab^	Nd
*CoB* (n=10)	3.76±2.30^b^	2.37±1.30^abc^	0.63±0.30^bc^	0.62±0.53^bc^	0.17±0.12^de^	0.03±0.04^ab^	Nd
*CoD* (n=10)	0.50±0.32^c^	0.52±0.27^bc^	4.71±2.60^a^	0.54±0.52^bc^	0.21±0.17^de^	0.04±0.01^ab^	Nd
*CoG* (n=10)	0.55±0.26^ab^	4.37±3.00^ab^	4.35±3.50^a^	0.86±0.64^abc^	0.26±0.12^cde^	0.04±0.07^ab^	Nd
*CoS* (n=10)	3.38±0.89^b^	5.20±2.70^a^	3.82±1.00^abc^	1.03±0.30^abc^	1.31±0.28^b^	0.08±0.07^ab^	Nd
*CoJ* (n=10)	7.60±1.50^a^	3.33±1.38^abc^	0.41±0.26^c^	0.81±0.64^bc^	0.16±0.12^de^	0.04±0.03^ab^	Nd
*GoD* (n=10)	4.99±2.70^b^	5.57±3.20^a^	4.18±3.00^a^	0.87±0.84^abc^	0.16±0.09^de^	0.03±0.03^ab^	Nd
*GoG* (n=10)	0.67±0.22^c^	3.09±1.10^abc^	4.81±2.96^a^	1.54±0.13^ab^	1.98±0.38^a^	0.06±0.04^ab^	Nd
*GoS* (n=10)	4.05±2.80^b^	5.72±3.60^a^	0.33±0.09^c^	0.82±0.70^bc^	0.57±0.25^c^	0.07±0.06^ab^	Nd
*GoJ* (n=10)	0.33±0.13^c^	0.53±0.28^bc^	3.27±2.95^abc^	1.13±1.02^abc^	0.43±0.50^cd^	0.10±0.12^a^	Nd
P-value	0.0000****	0.052 (NS)	0.016*	0.084 (NS)	0.0000****	0.405 (NS)	---

Values bearing different letters in a column are significantly different (p<0.05), NS=Not significant, TMF=Total microbial flora, LAB=Lactic acid bacteria, Y&M: Yeasts and molds, S. *aureus=Staphylococcus aureus*, TC=Total coliforms, TTC=Thermotolerant coliforms, SS=*Salmonella* or *Shigella*, Nd=Not detected

**Figure-3:**
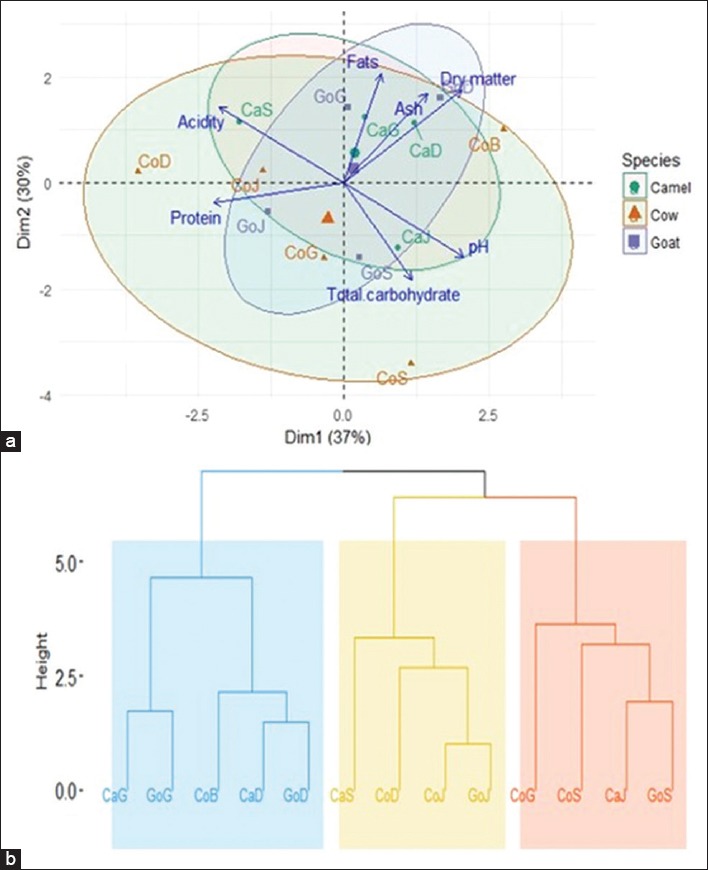
Principal component analysis distribution of fermented milk samples and ellipse of inertia different species on the factorial plane according to physicochemical parameters (a), ascending hierarchical clusters according to physicochemical parameters of fermented milk from different species (b).

**Figure-4:**
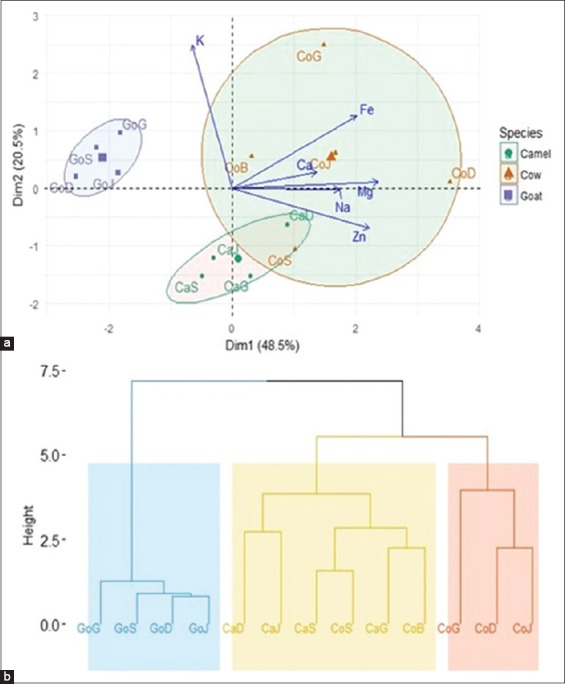
Principal component analysis distribution of fermented milk samples and ellipse of inertia different species on the factorial plane according to minerals concentration (a); ascending hierarchical clusters according to minerals concentration of fermented milk from different species (b).

Table-4 presents the average values of physicochemical parameters of fermented milk collected from different localities. A significant decrease in pH from 3.830 to 4.137 and a significant increase in acidity from 1.888 to 2.822 were found for fermented milk samples. The dry matters and ashes ranged, respectively, from 8.271 to 13.004 and from 1.994 to 4.761. Dry matter contents varied from 8.271% to 13.004% and ash values were significantly different between the samples collected from 0.199% to 0.476%. The biochemical composition of fermented milk samples varied as follows: Fats (1.210-3.863%), proteins (2.125-3.764%), and total carbohydrates (3.080-5.428%). Analyzing the distribution of center gravity classes on principal factorial plane (Figure-3a), it reveals a homogeneity of the groups of milk from different animal species according to the physicochemical parameters. Dimension 1 indicated that *CaG*, *CaD*, *GoG*, and *GoD* contain high rate of fats, ash, dry matter, and less rate of total carbohydrate, contrary to *CaJ*, *GoS*, *CoS*, and *CoG* who are rich total carbohydrate and poor in fats, ash, and dry matter. Dimension 2 reveals a high acidity, low pH, and low rate of protein samples content for the following *CaS*, *CoD*, *CoJ*, and *CoB* while *GoJ* is rich in protein and has high pH. The Ascending Hierarchical Clustering (AHC) led to a dendrogram which regrouped three major clusters according to their physicochemical parameters from different species (Figure-3b). The first cluster included the following samples *CaG*, *GoG*, *CoB*, *CaD*, and *GoD*. The second cluster included the fermented milk samples *CaS*, *CoD*, *CoJ*, and *GoJ*, and the third cluster contained the samples *CoG*, *CoS*, *CaJ*, and *GoS*. Table-5 shows that the mean values of major elements (Ca^2+^, Na^2+^, and K^+^) in fermented milk were 855.430 (*CaJ*), 424.296 (*CaG*), and 1427.383 (*CoG*) while the mean values of some minor elements (Fe^2+^, Zn^2+^, and Mg^2+^) were 4.421 (*CoJ*), 7.450 (*CoD*), and 104.941 (*CoD*), respectively. The Na^2+^/K^+^ and Na^2+^/K^+^ ratios obtained for the different fermented milk were ranged 0.104-0.909 and 3.392-6.464, respectively. Analyzing the distribution of centers gravity classes on the main factorial plane (Figure-4a), we can observe that camel milk with cow milk was close but goat milk deviated by its composition. The result of principal component analysis performed on the minerals concentration of different fermented milk samples showed that the first two axes explained 69.0% of the variation observed (Figure-4a). Therefore, only the first two axes were used to describe the relationship between mineral concentration and species samples. Dimension 1 shows that *CoS*, *CoB*, *CaG*, and *CoG* were poor in K^+^, but *CaS* and *CaJ* were highly rich in K^+^. Dimension 2 reveals that *CaD* was rich in Zn^2+^ and Na^2+^ and poor in Fe^2+^, Ca^2+^, and Mg^2+^, while *CoJ*, *CoD*, *GoD*, *GoS*, *GoJ*, and *GoG* were rich in Fe^2+^, Ca^2+^, and Mg^2+^ but poor in Zn^2+^ and Na^2+^. The AHC led to dendrogram which regroups three major clusters according to their minerals concentration (Figure-4b). The first cluster included the fermented milk samples from goat. The second cluster included the fermented milk samples from camel and cow (Bobo and Sebba). The third cluster contained the fermented cow’s milk samples from the remaining localities (Djibo, Dori, and Gorom-Gorom).

**Table-4:** Physicochemical profile of different fermented milk samples.

Samples	pH	Acidity	Dry matter (%)	Ash (%)	Fats (%)	Protein (%)	Total carbohydrate (%)
*CaD* (n=4)	3.944±0.053^abc^	2.360±0.173^abc^	11.557±2.260^abc^	0.401±0.120^abc^	3.760±1.500^ab^	2.125±1.724^c^	5.271±3.670^ab^
*CaG* (n=6)	3.999±0.152^abc^	2.283±0.391^abc^	10.221±0.830^abc^	0.346±0.210^abcde^	2.839±2.000^ab^	3.189±1.710^bc^	3.847±1.067^ab^
*CaJ* (n=3)	4.070±0.117^abc^	2.092±0.249^bc^	10.506±0.611^abc^	0.312±0.230^bcdef^	2.197±1.063^ab^	3.284±2.700^bc^	4.713±1.720^ab^
*CaS* (n=3)	3.830±0.060^c^	2.822±0.223^a^	9.044±1.390^bc^	0.377±0.126^abcd^	2.276±0.843^ab^	2.259±2.141^bc^	4.132±3.305^ab^
*CoB* (n=3)	4.092±0.094^ab^	2.085±0.170^bc^	12.197±5.160^ab^	0.476±0.125^a^	3.560±1.100^ab^	3.695±2.291^ab^	4.466±1.033^ab^
*CoD* (n=4)	3.861±0.060^bc^	2.629±0.294^ab^	9.893±1.961^bc^	0.241±0.155^def^	2.124±2.010^ab^	3.764±3.310^a^	3.764±1.460^ab^
*CoG* (n=3)	3.960±0.101^abc^	2.345±0.287^abc^	8.271±2.700^c^	0.199±0.138^f^	2.450±2.000^ab^	2.278±1.052^bc^	3.334±1.043^ab^
*CoJ* (n=3)	3.940±0.150^abc^	2.408±0.460^abc^	9.145±2.063^bc^	0.330±0.115^abcdef^	2.170±0.998^ab^	3.392±1.330^bc^	3.253±1.272^ab^
*CoS* (n=3)	4.137±0.118^a^	1.888±0.314^c^	8.746±0.822^bc^	0.222±0.210^ef^	2.192±1.500^ab^	2.287±2.116^bc^	4.045±1.345^ab^
*GoD* (n=5)	3.988±0.160^abc^	2.353±0.510^abc^	13.004±3.433^a^	0.463±0.180^ab^	3.720±1.651^ab^	3.690±0.715^ab^	5.131±1.720^ab^
*GoG* (n=4)	4.065±0.362^abc^	2.278±0.470^abc^	10.817±2.674^abc^	0.274±0.100^cdef^	3.863±0.932^a^	3.600±0.533^ab^	3.080±1.320^b^
*GoJ* (n=3)	3.953±0.050^abc^	2.376±0.200^abc^	9.236±2.332^bc^	0.320±0.101^bcdef^	2.146±2.082^ab^	2.534±1.314^bc^	4.236±2.063^ab^
*GoS* (n=3)	4.000±0.110^abc^	2.308±0.322^abc^	9.148±1.460^bc^	0.285±0.184^bcdef^	1.210±1.140^b^	2.225±1.100^bc^	5.428±2.360^a^
p-value	0.440 (NS)	0.225 (NS)	0.222 (NS)	0.010*	0.688 (NS)	0.050 (NS)	0.610 (NS)

Values bearing different letters in a column are significantly different (p<0.05), NS=Not significant, Dry matter=Total solids

**Table-5:** Concentrations in mineral of different samples (mg/Kg).

Samples	Ca^2+^	Mg^2+^	Na^2+^	K^+^	Fe^2+^	Zn^2+^	Na^2+^/K^+^	Ca^2+^/Mg^2+^
*CaD* (n=4)	664.664±0.050^bc^	103.437±0.002^ab^	285.176±0.000^abcd^	658.739±0.001^bcd^	1.280±0.199^c^	3.678±0.048^bcd^	0.433	6.426
*CaG* (n=6)	270.449±0.000^c^	71.701±0.003^abc^	424.296±0.012^a^	466.970±0.001^d^	1.222±0.429^c^	4.081±0.331^bc^	0.909	3.772
*CaJ* (n=3)	855.430±0.040^a^	50.330±0.001^c^	246.182±0.003^abcd^	474.310±0.000^d^	1.135±0.519^c^	3.713±0.462^bcd^	0.519	16.996
*CaS* (n=3)	239.009±0.004^c^	70.456±0.005^abc^	201.042±0.004^bcd^	487.816±0.000^d^	1.338±0.000^c^	4.701±0.005^bc^	0.412	3.392
*CoB* (n=3)	362.085±0.031^bc^	70.882±0.001^abc^	358.986±0.001^abc^	1083.825±0.001^abc^	2.108±0.620^bc^	4.455±0.000^bc^	0.331	5.108
*CoD* (n=4)	686.440±0.060^ab^	104.941±0.002^a^	297.282±0.000^abcd^	714.067±0.001^bcd^	4.421±0.250^a^	7.450±0.058^a^	0.416	6.541
*CoG* (n=3)	703.008±0.001^ab^	93.635±0.000^abc^	393.185±0.001^ab^	1427.383±0.000^a^	3.527±1.001^ab^	3.104±0.167^bcd^	0.275	7.508
*CoJ* (n=3)	361.406±0.016^bc^	92.330±0.000^abc^	252.075±0.003^abcd^	851.585±0.001^bcd^	4.259±0.000^a^	5.341±0.053^ab^	0.296	3.914
*CoS* (n=3)	323.614±0.020^c^	93.260±0.000^abc^	267.594±0.015^abcd^	529.166±0.000^cd^	2.244±0.004^bc^	5.216±0.424^ab^	0.505	3.470
*GoD* (n=5)	289.084±0.003^c^	47.587±0.001^c^	147.065±0.001^d^	950.956±0.000^abcd^	1.191±0.401^c^	1.760±0.089^d^	0.155	6.075
*GoG* (n=4)	343.123±0.001^c^	53.078±0.000^c^	126.038±0.000^d^	1207.313±0.000^ab^	1.777±0.129^bc^	3.146±0.095^bcd^	0.104	6.464
*GoJ* (n=3)	329.874±0.036^c^	53.580±0.000^bc^	161.165±0.020^cd^	1002.359±0.000^abcd^	1.359±0.006^c^	2.829±0.006^bcd^	0.161	6.157
*GoS* (n=3)	216.119±0.019^c^	50.650±0.000^c^	191.068±0.024^bcd^	1140.290±0.000^ab^	1.414±0.115^c^	2.274±0.404^cd^	0.158	4.267
p-value	0.004**	0.136 (NS)	0.055 (NS)	0.014*	0.001**	0.010*	----	----

Values bearing different letters in a column are significantly different (p<0.05), NS=Not significant, Ca^2+^=Calcium, K^+^=Potassium, Na^2+^=Sodium, Mg^2+^=Magnesium, Fe^2+^=Iron, Zn^2+^=Zinc

## Discussion

**Figure-5:**
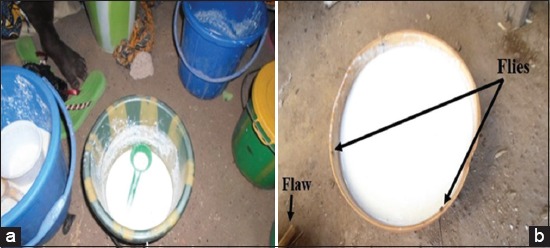
Milk sale in Dori (a) and Gorom-Gorom (b) market.

The quality and safety of fermented foods are decisive factors for producers and consumers. Microbial densities obtain during this study were higher than those reported by Bonfoh *et al*. [16], Koussou *et al*. [17], Katinan *et al*. [18], and De *et al*. [19]. Total microbial flora in samples was high according to the criteria presented in Codex Standard for Fermented Milks which lays down the minimum sum of microorganisms 10^7^ CFU/ml. The presence of high densities of microorganisms in these samples could be due to poor handling, inadequate heat treatment, and environmental conditions during the preparations and sale. As for the lactic acid bacteria, these results did not reflect poor quality of milk but more contributing to the improvement of its sanitary quality, because these microorganisms produce antibacterial substances and particular organic acids [20-24]. Yeasts are responsible for producing alcohol during the fermentation of milk. These results were similar to those reported by Savadogo *et al*. [25], Al-Tahiri [26], and Serhan and Mattar [27]. The assessment of hygienic quality detected the presence of thermotolerant coliforms and *S. aureus* in fermented milk. Several studies have shown the presence of coliforms. These results obtained are in agreement with the finding of Katinan *et al*. [18] on fermented milk produced and consumed in Yamoussoukro town (Ivory Coast). This presence of microorganisms would result due to the processing environment, the sale condition, and the lack of hygienic measures during milk handling [6]. Figure-5 illustrates the conditions in which the traditional fermented milks are sold in Burkina Faso (Dori and Gorom-Gorom). The presence of suspected pathogens (*S. aureus* and coliforms) reflects the lack of knowledge of certain rules of good hygiene and production practices by dairy producers in these areas. This absence would be explained by the fact that analyzed milk did not provide from sick or carrying animals and was not contaminated by individuals carrying or sick during the sale. The poor quality fermented milk, unclean, and insufficient cleaning of milk equipments were among the most important sources of milk contamination. The milk is generally exposed to different contaminants when it transferred from one container to another, transported to consumers as well as retailers from the production site without cooling facilities, and with no proper milk containers [28,29]. In general, these high rates of microorganisms can be explained by the fact that traditional production is neither controlled nor regulated, as the majority of producers and distributors are not sufficiently informed about hygiene and risk management measures [6,29,30]. The quality of fermented milk and yogurt is determined by several factors such as the composition and the microbiological quality of materials (raw milk and the added ingredients), the preparation and processing of milk, and the manipulation of the clot after fermentation [31].

The quality of fermented milk depends on good quality of the raw material and efficient control at all processing stages. These results may be attributed to the presence of carbohydrates in milk stimulating the growth of lactic acid bacteria during the fermentation. The pH and acidity obtained are similar to those reported on fermented milks in Burkina Faso [6], China [32], Chad [33] and United Arab Emirates [34]. This acidity is related to the metabolic activity of the lactic microbiota in the fermented milk. The level of lactic acid depends on the amount of fermentable sugar and milk protein hydrolyzed by these bacteria [31]. According to Savadogo *et al*. [20], low pH values prevent the growth of most spoilage and pathogenic organisms but create a suitable environment for the growth of probiotic organisms (lactic acid bacteria, yeasts, and molds). The composition out of dry matter, fats, proteins, total carbohydrates, and ash content varies from a sample to another. These results are similar with those reported by Wang *et al*. [32] in fermented milk of goats in China, which were 13.02, 2.90, 3.50, and 5.97 for dry matter, fats, proteins, and total carbohydrates respectively, but a rate of ash (0.75) different in this study (0.476). Semaan *et al*. [35], Cesbron-Lavau *et al*. [36], and Ayyash *et al*. [34] obtained similar values from traditional dairy products (milk and darfieh cheese), but other compositions are reported in review by Clark and Mora-Garcia [37]. Numerous studies reported that fermented milk and yogurt contain some nutritive components such as peptides and fatty acids, which are produced during fermentation. These components were known to modulate the immune system [38]. Yadav and Shukla [39] reported that fermented milk consumption could prevent the effect of ulcerative colitis. Camel milk differs from other milk (bovine) in its composition and protein content and structure, and, therefore, is expected to possess functional and bioactive properties different from bovine milk. Camel milk has an excellent reputation as nutritious food, with most of its therapeutic value related to its biological properties such as antioxidant activity [7].

Table-5 showed the average contents of minerals in various traditional fermented milks of Burkina Faso. The results revealed that camel fermented milk contains the highest concentration in Na^2+^ and Ca^2+^. High concentrations of Ca^2+^, Mg^2+^, and Zn^2+^ were reported by Navarro-Alarcón *et al*. [39] in commercial fermented milk of goat and cow, but Wang *et al*. [32] reported high concentrations to K^+^ (1724 mg/Kg) and Ca^2+^ (1409 mg/Kg) in fermented goat milk. The concentration of Ca^2+^, K^+^, and Mg^2+^ in fermented milks could be due to the activity of the lactic bacteria during fermentation process [40,41]. The high rate of Ca^2+^ in milk of Djibo, Dori, and Gorom-Gorom is due to the high presence of limestone (CaCO_3_) in the water of these cities. In general, the minerals concentration in fermented milk depends on the species, its individual characteristics, feeding method, rearing area, nature of metal of the material containing milk, degree of food contamination and drinking water, lactation stage, and health condition of female. The Na^2+^/K^+^ ratio in the body helps to control blood pressure; fermented milk is a food source having impact in lowering blood pressure [42]. The Na^2+^/K^+^ ratios (0.104-0.909) were obtained for the different fermented milk samples, and this low Na^2+^/K^+^ ratio can help to control blood pressure. The Ca^2+^/Mg^2+^ ratio (3.392-6.464) for food was within the recommended value higher at 1.00 [42]. The fermented milk samples were a rich source of Ca^2+^ and Mg^2+^. K^+^ and Ca^2+^ are the most important elements for bone growth, development, metabolism, and health maintenance.

## Conclusion

The traditional fermented milks and dairy products are important sources of functional nutrients. The fermented milks sold and consumed in Burkina Faso shown high variability in microbiological, physicochemical quality, and the minerals concentration. This study revealed that traditional fermented milk is a very important source of nutrients and functional food due to compounds in fats, proteins, carbohydrate, and low Na^2+^/K^+^ ratio. The presence of lactic acid bacteria and yeast improves the organoleptic qualities of fermented milk and brings beneficial effects to consumers, while the presence of certain bacteria such as coliforms and *S. aureus* is a risk for the milk quality and the health of consumers. This work has important implications for the commercialization of fermented milk based on camel and goat milk. The sanitary practices followed by producers during handling, storage, and processing are generally poor. Based on the overall evaluation of the results, training to the local producers on good hygiene practices is necessary to improve sanitary quality of fermented milk sold in Burkina Faso.

## Authors’ Contributions

HC collected the samples and wrote the original draft of the manuscript. HC, JUM, and SMD analyzed the samples. NSS, AS, and FT organized the data, helped in writing, and review of the manuscript. CZ, YT, and AlyS supervised the study, validated the results from analysis, and review of the manuscript. All authors have read and approved the final version.
